# Airway microbiome-immune crosstalk in chronic obstructive pulmonary disease

**DOI:** 10.3389/fimmu.2022.1085551

**Published:** 2023-01-17

**Authors:** Alex Kayongo, Nicole M. Robertson, Trishul Siddharthan, Moses Levi Ntayi, Josephine Caren Ndawula, Obondo J. Sande, Bernard S. Bagaya, Bruce Kirenga, Harriet Mayanja-Kizza, Moses L. Joloba, Sofia K. Forslund

**Affiliations:** ^1^ Makerere University Lung Institute, Makerere University College of Health Sciences, Kampala, Uganda; ^2^ Department of Medicine, College of Health Sciences, Makerere University, Kampala, Uganda; ^3^ Department of Immunology and Molecular Biology, College of Health Sciences, Makerere University, Kampala, Uganda; ^4^ Department of Medicine, Center for Emerging Pathogens, Rutgers, The State University of New Jersey, New Jersey Medical School, Newark, NJ, United States; ^5^ College of Medicine, University of Kentucky, Lexington, KY, United States; ^6^ Division of Pulmonary Medicine, School of Medicine, University of Miami, Miami, FL, United States; ^7^ Experimental and Clinical Research Center, Max Delbrück Center for Molecular Medicine in the Helmholtz Association, Berlin, Germany; ^8^ Experimental and Clinical Research Center, a cooperation of Charité - Universitatsmedizin Berlin and Max Delbrück Center for Molecular Medicine, Berlin, Germany; ^9^ Charité-Universitatsmedizin Berlin, corporate member of Freie Universität Berlin, Humboldt-Universität zu Berlin, and Berlin Institute of Health, Berlin, Germany; ^10^ DZHK (German Centre for Cardiovascular Research), Partner Site Berlin, Berlin, Germany; ^11^ Structural and Computational Biology Unit, European Molecular Biology Laboratory, Heidelberg, Germany

**Keywords:** COPD, lung microbiome, mucosal immunity, inflammation, innate immunity, adaptive immunity

## Abstract

Chronic Obstructive Pulmonary Disease (COPD) has significantly contributed to global mortality, with three million deaths reported annually. This impact is expected to increase over the next 40 years, with approximately 5 million people predicted to succumb to COPD-related deaths annually. Immune mechanisms driving disease progression have not been fully elucidated. Airway microbiota have been implicated. However, it is still unclear how changes in the airway microbiome drive persistent immune activation and consequent lung damage. Mechanisms mediating microbiome-immune crosstalk in the airways remain unclear. In this review, we examine how dysbiosis mediates airway inflammation in COPD. We give a detailed account of how airway commensal bacteria interact with the mucosal innate and adaptive immune system to regulate immune responses in healthy or diseased airways. Immune-phenotyping airway microbiota could advance COPD immunotherapeutics and identify key open questions that future research must address to further such translation.

## Introduction

Chronic Obstructive Pulmonary Disease (COPD) has been characterized by persistent respiratory symptoms and airflow limitation due to distal airway abnormalities (1). This is usually caused by exposure to noxious particles or gases and is influenced by several host factors (1). Chronic airway inflammation drives small airway changes and destruction of lung parenchymal tissue (2–5). Individuals diagnosed with COPD have varying degrees of chronic bronchitis, distal airway disease, and parenchymal destruction (6). COPD is the third leading cause of death worldwide, with several epidemiologic studies reporting a global prevalence of approximately 11.7% (95% CI: 8.4-15%) (1). According to recent projections, the prevalence is expected to rise over the next 40 years, with approximately 5 million people succumbing to COPD-related death annually (1). The prevalence in Africa has been reported as overall similar to other regions (1, 7).

The mechanisms driving COPD progression have yet to become fully known. The airway microbiome has been implicated in several respiratory diseases, such as COPD, bronchiectasis, and asthma (8). However, it is still unclear how changes in airway microbiota drive persistent immune activation and consequent lung damage in COPD. Whereas *N. subflava* has been recently demonstrated to drive bronchiectasis, only indirect inference is presently possible for COPD (9, 10). Because of the existence of a well-documented overlap between COPD and bronchiectasis (11–15), it is plausible that *N. subflava* could contribute to COPD pathogenesis. This, however, needs further investigation. Several studies published elsewhere have also described the airway microbiome in COPD and health (16–30). Dysbiosis (defined as an unhealthy microbial compositional state) in the airways among COPD patients has been associated with disease progression and poor outcomes (23, 29, 31–37). Immune activation of the airways drives COPD progression (38–46). However, it is still unknown how dysbiosis fuels such persistent airway immune activation in COPD. In this review, we examine how dysbiosis may mediate COPD-associated airway inflammation. Although the mechanisms of airway microbiome-immune crosstalk have not been thoroughly investigated, we give a detailed account of how airway commensal bacteria interact with the mucosal innate and adaptive immune system to regulate immune responses in diseased airways. Furthermore, borrowing a page from gut microbiome-immune interactions published elsewhere (47–53), we suggest possible mechanisms worth investigating that could be contributing to COPD disease.

Upon microbial interaction with mucosal immune cells, metabolic and epigenetic changes occur (54–66), inducing immunologic tolerance aimed at minimizing damage potentially arising from responses against invading bacteria (67–69). Several researchers have highlighted the salience of microbiome-mediated immune regulation (50, 70–78). One of the earliest pieces of evidence for this immune regulation was the observation of gene reprogramming following colonization with a bacterial commensal in germ-free mice (79). Such changes are mediated *via* the activity of bacterial metabolites, discussed in detail in a separate section in this review. Remarkably, most of them (62, 80) attenuate pro-inflammatory responses *via* epigenetic changes in immune cells, inducing a switch from transcriptionally active to silent chromatin states (67). For instance, butyrate, a short-chain fatty acid, suppresses the activity of NF-kB, consequently inhibiting the production of pro-inflammatory cytokines (81, 82). Similarly, ethionine suppresses the activity of NF-kB and TNFα following stimulation with lipopolysaccharide (LPS) (83), while lactate promotes histone acetylation at the *IL10* promoter, enhancing *IL10* transcription in macrophages (84). The metabolite deoxycholate alters H3K4me3 and H3K27me3 in bone marrow granulocyte progenitor cells, leading to neutrophil proliferation (85). In germ-free and antibiotic-treated mice, dendritic cell activation and the consequent production of type 1 interferons is impaired, a response driven by reduced levels of H3K4me3 on transcriptional start sites of pro-inflammatory response factors *irf-3* and -*7* (86). Immune reprogramming has also been noted among adaptive immune cells. Treg cells are induced *via* the activity of HDAC at the locus of the *Foxp3* transcription factor (87–89). In contrast, Th17 differentiation is inhibited (90–92). Comparative analysis of metabolites shows a differing degree of HDAC inhibition, epigenetics, and immune functional consequences (80).

**Figure 1:**
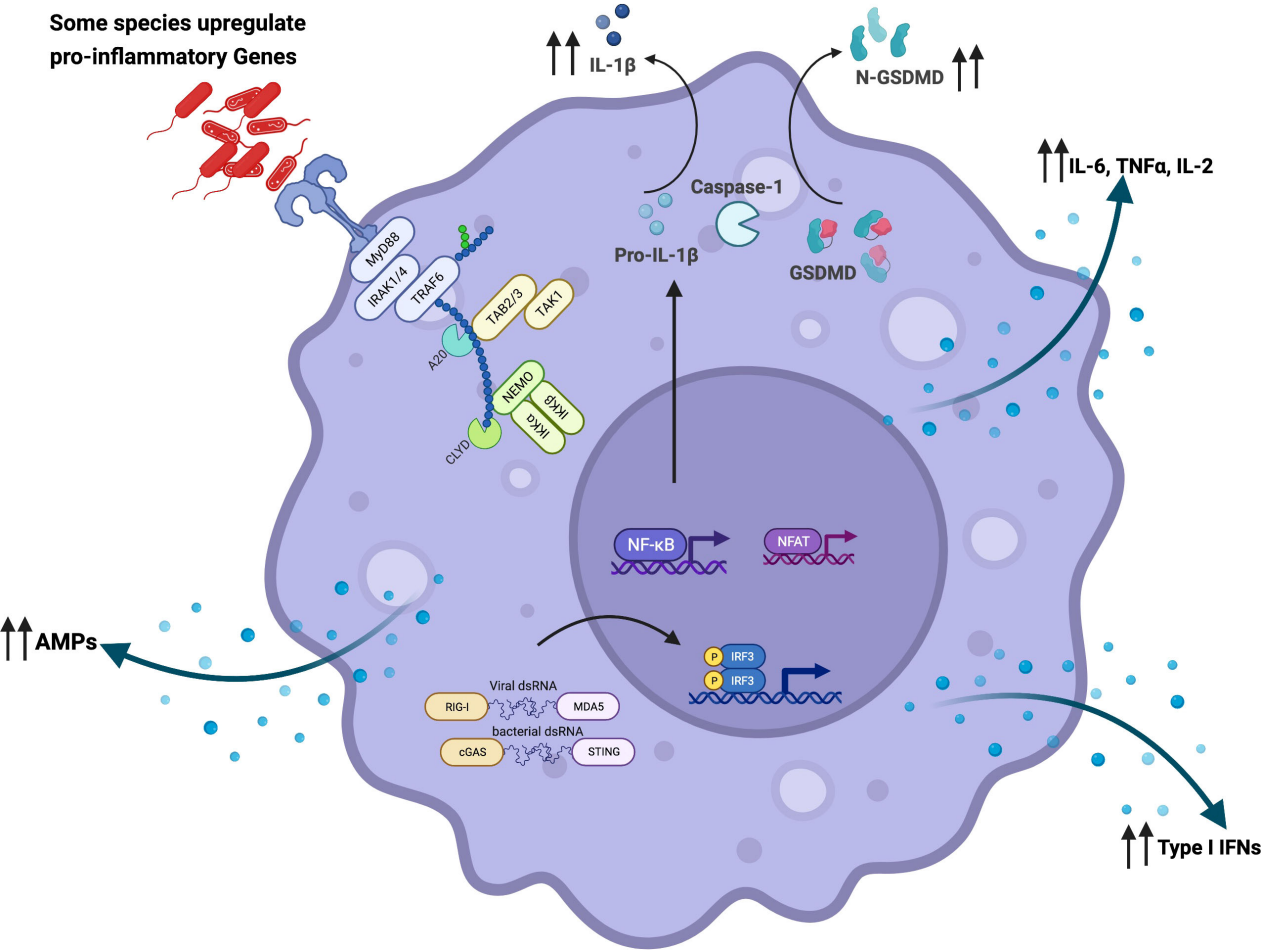
Microbial interaction with the innate immune system activates and reprograms immune cells: Airway microbiome-derived products such as lipopolysaccharide are detected by surface and intracellular immune sensors such as Toll-like receptors (TLRs). In a MyD88-dependent manner, airway epithelial cells, alveolar macrophages, and dendritic cells become activated and upregulate the expression of pro-inflammatory cytokines such as IL-1β, IL-18, TNFα, IL-6, and IL-2. These activated cells orchestrate immune inflammation. Microbicidal activity of alveolar macrophages is also potentiated through increased gene expression of antimicrobial peptides and other lytic proteins. The microbiome has also been shown to potentiate macrophage-bacterial killing and clearance *via* GM-CSF signaling. Furthermore, depletion of commensal bacteria has been shown to reprogram alveolar macrophages from classical (M1) to alternative (M2) phenotype characterized by the expression of higher levels of Arg1, CCL24, IL-13, IL-10, IL-6, and IL-1β. In addition to MyD88-dependent activation, intracellular sensing of microbial-derived products by NOD1, NOD2, and NLRP6 in the epithelial cells set the pace for a pro-inflammatory signal along the mucosa. This restricts mucosal colonization by pathogenic bacteria *via* the secretion of IL-1β and IL-18. Microbiome-driven-type-I-IFN responses dependent on cGAS-STING activation have also been described. Created with BioRender.com.

**Figure 2:**
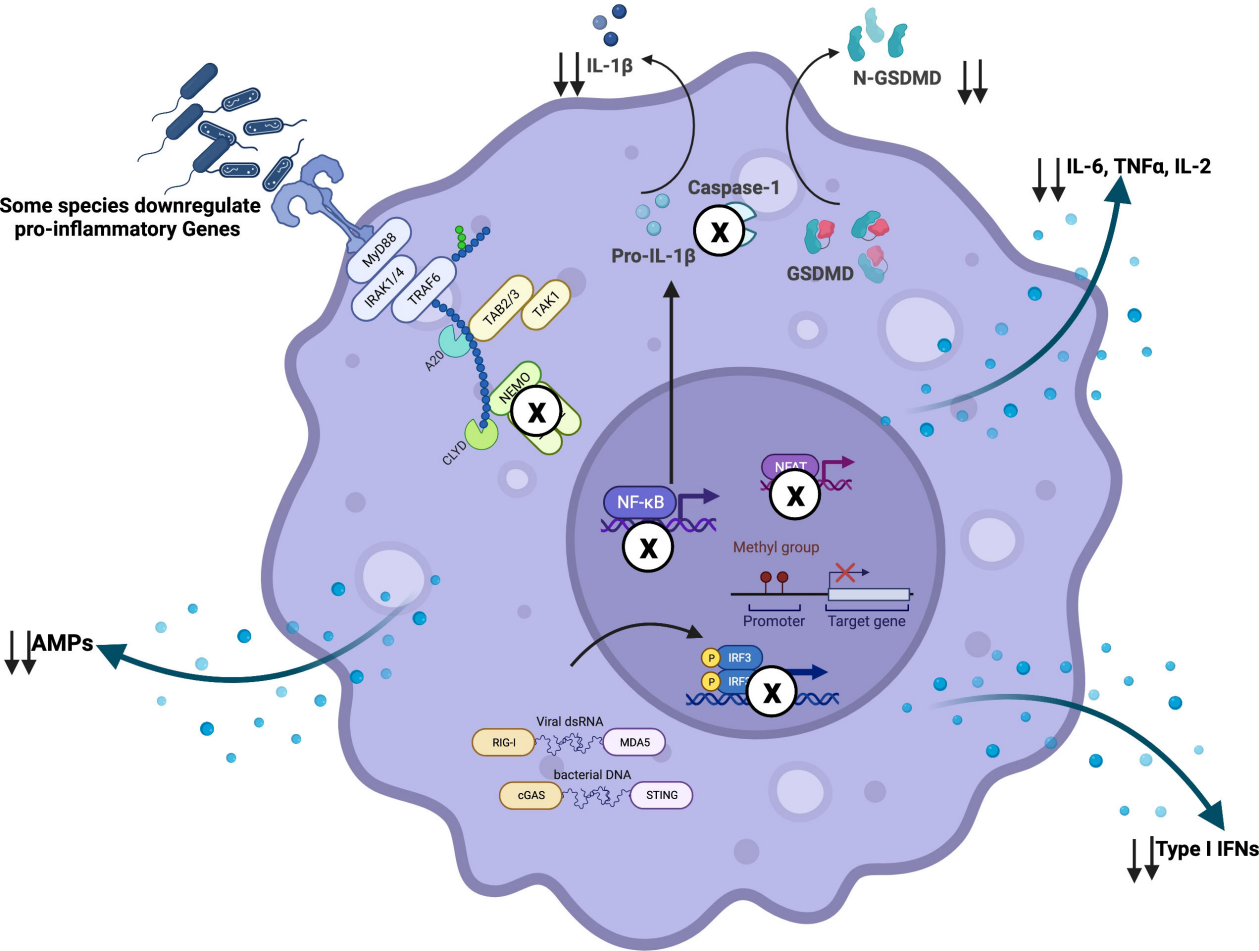
Airway microbiome-driven immunomodulation: Airway microbiome downregulates pro-inflammatory signals *via* interference with immune signaling machinery. For instance, *R. mucilaginosa* has been shown to inhibit immune activation *via* NF-κB dependent mechanisms. As an immune evasion strategy, pathobionts have been described to inhibit TRIF and NFAT signaling, phagolysosomal fusion and acidification, as well as inflammasome activation. In other scenarios, gene methylation may promote transcriptional repression of pro-inflammatory genes. Created with BioRender.com.

Epigenetic modifications in germ-free mice have been characterized by methylation patterns on inflammatory genes such as those encoding Toll-like receptors, chemokines, and cytokines (93). In neonatal mice, the microbiome induces decreased methylation of the chemokine-encoding gene *Cxcl16*, which is critical in recruiting iNKT cells into the mucosa (94). Consequently, this change ameliorates inflammation in the gut and the airway mucosa (94). In another scenario, comparative epigenetic analysis of myeloid cells, derived from microbiome-colonized germ-free mice, shows a trimethylation pattern of histone H3 at lysine 4 of the loci of pro-inflammatory genes such as the genes encoding type 1 interferons, which as a result inhibits pro-inflammatory signals (95). Indeed, the role of epigenetics in mucosal immunity has been confirmed by a resultant loss in barrier integrity following the deletion of the *histone deacetylase-3* gene from epithelial cells (96). In a nutshell, these findings support the role of microbiota in reprogramming mucosal immune cells, as illustrated in **Figures 1**, **2**. Although the gut microbiome in these studies provides reprogramming signals, available studies further implicate airway microbiome initiating and mediating immune reprogramming locally at the mucosa. As supporting evidence, intranasal administration of a bacterial lysate abrogates experimental allergic asthma by targeting dendritic cells, epithelial cells, and type 2 ILCs (97). Whether similar findings occur in the setting of COPD remains to be investigated.

**Figure 3:**
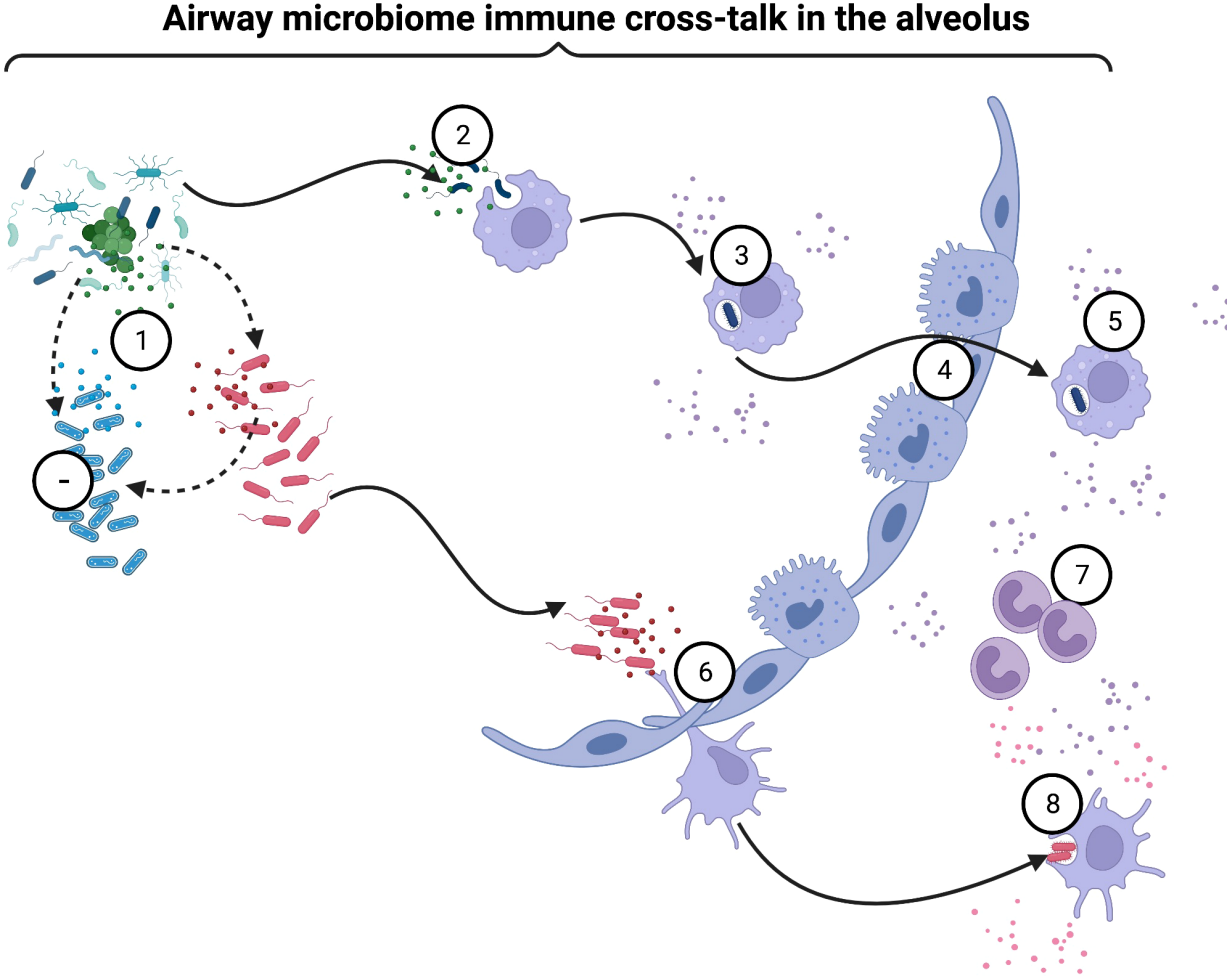
Airway microbiome immune crosstalk in alveoli: Following successful colonization of the respiratory tract, bacterial species replicate rapidly and establish dense biological communities (1). Some bacteria inhibit other species *via* the secretion of antimicrobial peptides and lytic enzymes(1). Alveolar macrophages engulf bacteria(2) and become activated *via* MyD88-dependent and -independent mechanisms(3). Airway microbiome sensing potentiates bacterial killing(3). Some intracellular pathobionts infect alveolar macrophages and access the lung interstitium (4). Activated macrophages secrete chemoattractants and promote the migration of other cellular players, such as monocytes, neutrophils, and adaptive immune response cells, into the airways to clear pathobionts (5). Dendritic cells (DCs) continuously sample airway bacteria that attach and colonize the mucosa *via* protruding dendrites(6). Airway microbiome sensing by bronchial epithelial cells, alveolar macrophages, and DCs activate innate lymphoid cells (ILCs) (7), which modulate other immune cells’ activity. Depending on the type and degree of microbial exposure, DCs induce a wide range of immune responses, from immune tolerance induced by plasmacytoid DCs (pDCs) to inflammation induced by conventional DCs (cDCs)(8). Continuous microbial sampling and trafficking by activated DCs, and alveolar macrophages deliver processed microbial antigens to naïve CD4+T and CD8+T cells within mucosa-associated lymphoid tissue (MALT) and draining lymph nodes (8). Created with BioRender.com.

**Figure 4:**
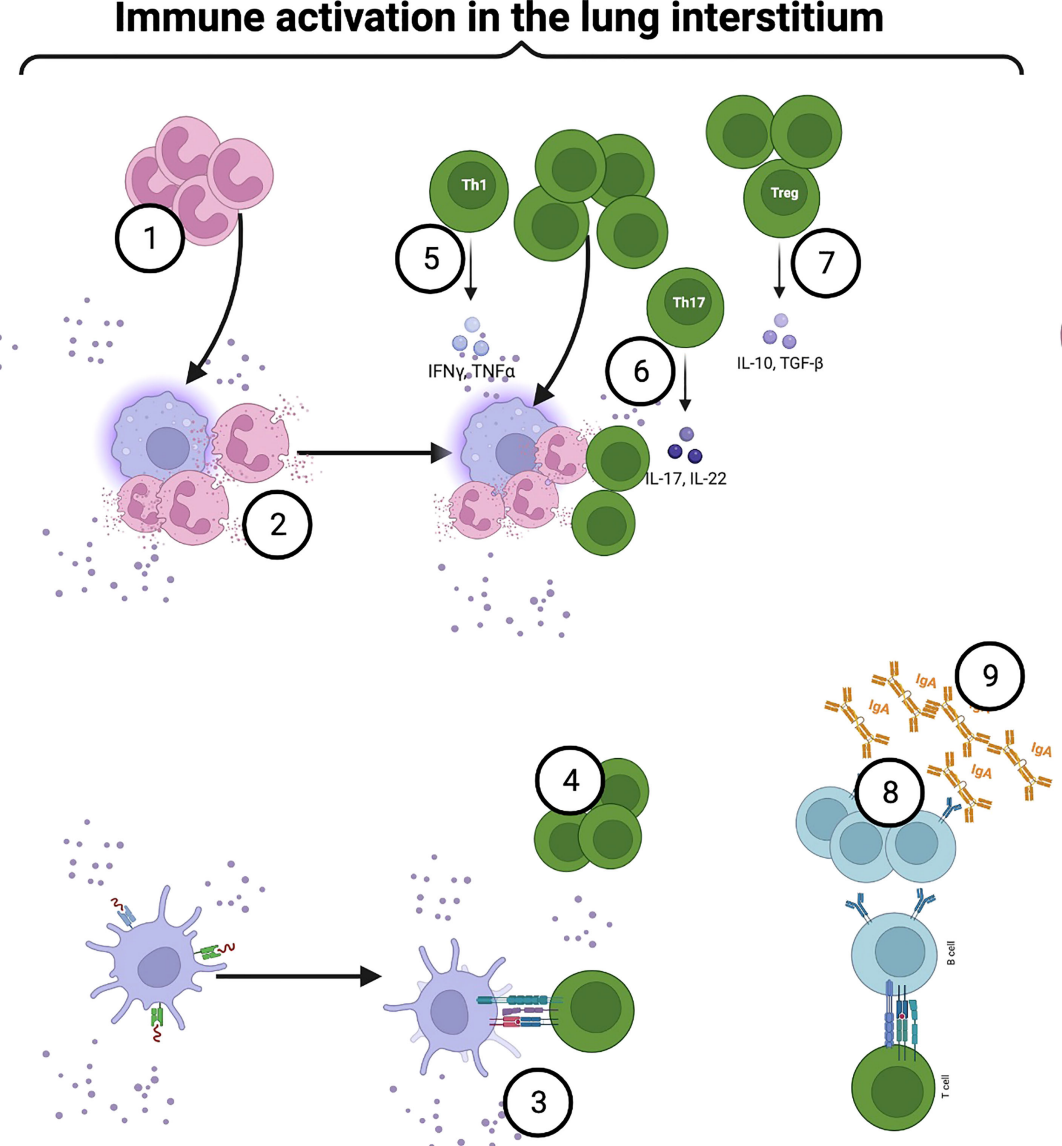
Airway microbiome immune crosstalk in the lung interstitium: Some intracellular pathobionts infect alveolar macrophages and access the interstitium. Activated macrophages secrete chemoattractants and promote neutrophilic infiltration into the airways to clear pathobionts (1). Upon bacterial sensing, neutrophils become activated, degranulate and release NETs (2). Continuous sampling and trafficking by activated DCs, and alveolar macrophages deliver processed microbial antigens to naïve CD4+T within mucosa-associated lymphoid tissue (MALT) and draining lymph nodes (4). Under a steady state, mature lung cDCs are preferentially programmed to induce a Th2 immune response. However, following immune sensing and activation, the production of IL-12, IL-23, IL-27, and notch ligand by airway DCs, alveolar macrophages, and epithelial cells induce a Th1 response (5). Among CD4+T cell phenotypes, microbiome-mediated mucosal inflammation has been strongly linked to aberrant Th17 (6). Besides the Th17 phenotype, microbial interaction with mucosal CD4+T cells induces immune tolerance (7). MyD88-dependent TLR2 activation by capsular polysaccharide A induces the expansion of Foxp3+T cells within the mucosa (7). Foxp3+T cells drive IL-10 production, facilitating mucosal immune tolerance (7). In another mechanism, microbial-induced Tregs promote mucosal memory B or plasma cells’ IgA secretion (8). Created with BioRender.com.

**Figure 5:**
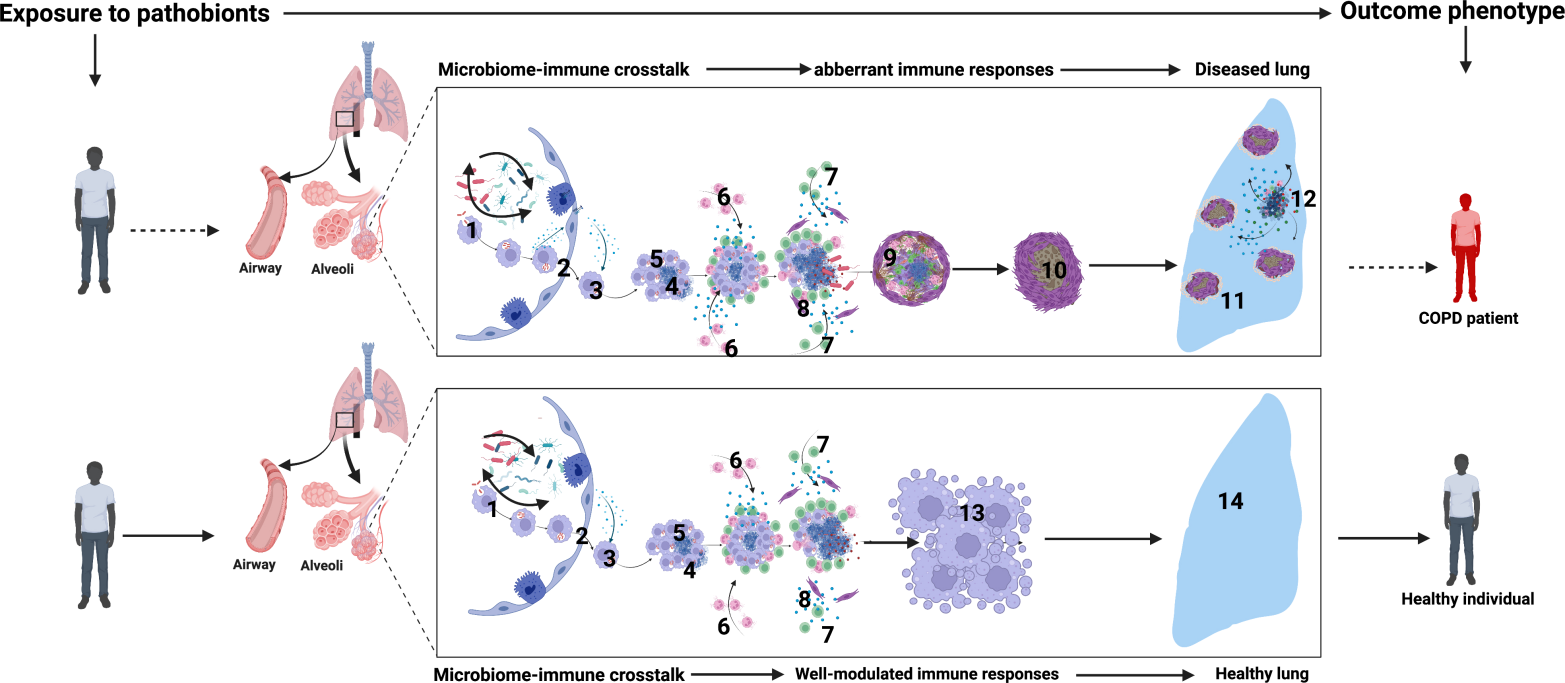
Airway microbiome-immune crosstalk in healthy versus diseased lungs (COPD): 1. Initial crosstalk between airway microbial communities and the mucosal innate and adaptive immune cell activation sets the tone of airway mucosal immune responses. 2. Infected macrophages from the distal airways and alveolar spaces migrate into the interstitium in an IL-1R-dependent manner. 3. Intracellular bacteria replicate within macrophages. 4. Some intracellular pathobionts induce infected macrophage apoptosis and expression of host lytic proteins in an ESX-1-dependent manner. 5. Newly recruited macrophages engulf infected cell debris. 6. Neutrophils infiltrate the airways to orchestrate inflammation, engulf dying infected cells, and kill bacteria through NETosis and the release of lytic enzymes. 7. Microbial-specific T cells arrive in airways and produce cytokines such as IFN-γ, which enhance the microbicidal activity of alveolar macrophages, monocytes, and DCs 8. Macrophage and neutrophil necroptosis lead to the release of lytic proteins such as MMPs, defensins, and cathepsin G into the extracellular space. Subsequent induction of lytic proteins causes extensive tissue damage. 9. Over time, with attempted healing and subsequent inflammation, extensive lung damage, fibrosis, and reduced lung function ensue. 10-11. Extensive fibrosis further reduces lung compliance and worsens lung function. 12. Sustained dysbiosis driven by periodic insults such as bacterial, viral, and fungal infections and air pollution or smoking orchestrate more damage and susceptibility. 13. In contrast, in healthy airways, robust innate and adaptive immune reprogramming promotes a highly modulated immune microenvironment with balanced lethal and resolving inflammatory states. This is optimal to contain airway commensals and pathobionts hence maintaining healthy lungs seen in 14. Created with BioRender.com.

Mechanistically, microbiome-derived signals such as lipopolysaccharide (LPS) and bacterial DNA stimulate alveolar macrophages and dendritic cells *via* surface and intracellular sensors, producing several activating signals (chemokines and cytokines) (**Figure 3**). In the next section, we elaborate on such responses and describe how they activate other immune cellular players, i.e., innate lymphoid cells, γδ-T cells, neutrophils, monocytes, and lymphocytes (**Figure 4**). A well-coordinated immune response from this cellular network maintains a robust immune barrier at the respiratory mucosa, which preserves the microbial ecology (98, 99). In diseased airways, however, repeated insults such as respiratory infections, cigarette smoke, and particulate matter alter microbiome composition, reducing microbial diversity (100). Consequently, the ensuing dysbiosis triggers immune activation, potentially propagating inflammation and tissue damage observed in COPD (**Figure 5**) (101, 102). Several observations highlighting this concept have been published. They are summarized here as follows. Infection of distal airways during early childhood with pathogenic bacteria has been reported to cause severe lung damage associated with impaired lung growth and a consequent reduction in lung function (FEV_1_/FVC) in adult life (103–105). Although these studies do not establish specific bacterial species orchestrating this damage, several studies report organisms such as *S. pneumoniae*, *H. influenzae, M. catarrhalis, S. aureus, and K. pneumoniae* as culprits (106–110). Besides bacterial-induced early childhood-lung injuries and associated COPD in adult life, an interesting hypothesis termed the vicious circle was described, stating that once bacterial pathogens have successfully colonized the airways following impaired mucociliary clearance secondary to a primary insult, they persist, inducing chronic airway inflammation (111). Following the publication of early reports in 2010 describing the lung microbiome (112–115), authors revised the vicious circle to suggest that insults such as tobacco smoke exposure, which impair airway mucosal defenses mediate dysbiosis, leading to dysregulated immune response, further impairment of mucosal defense and ensuing dysbiosis, inducing inflammation and further dysbiosis (99). In distal airways, this inflammation contributes to tissue damage and progressive obstruction, as is the case for *P. aeruginosa* and *N. subflava* (9, 116, 117). These effects are mediated *via* the release of bacterial outer membrane vesicles harboring extracellular products, such as LPS, a potent inflammatory stimulus demonstrated by earlier studies to induce lung emphysema in hamsters (118). Several COPD human studies have supported this hypothesis, showing that bacterial colonization of the airways induces inflammation (111, 115–117, 119–121) and impairs lung function (FEV_1_) (122).

## Airway bacterial colonization

Airway mucosa houses niche-specific bacterial communities varying in biomass density and diversity as one transition from the upper to lower respiratory tract (123). A physiological gradient primarily drives this difference as a consequence of a gradual increase in the relative humidity (124) and temperature (125), a decrease in the partial pressure of oxygen (pO_2_), an increase in the partial pressure of carbon dioxide (pCO_2_), and a gradually increasing pH along the respiratory tract (126, 127). pO2/pCO2 gradient is further determined by airway ventilation and the environmental air quality (126, 127). These physiological parameters determine niche-specific selective growth conditions that ultimately shape the microbial communities along the respiratory tract. Therefore, changes in airway physiological conditions directly impact airway microbial composition. During inhalation, bacteria-containing particles are deposited into the respiratory tract. Particles larger than 10μm in diameter remain in the upper respiratory tract, whereas particles smaller than 1μm in diameter reach the alveolar spaces (128). Pili and fimbriae facilitate the attachment of deposited bacteria on the respiratory mucosa (129). Most bacteria fail to attach to the mucosa and are cleared *via* mucociliary action of the respiratory tract (130); some become neutralized by mucosal surface IgA (131, 132) while others are broken down by mucosal antimicrobial peptides (133). The implication of these immune protective mechanisms in COPD is further elaborated in separate sections. Successfully attached bacteria colonize the airway mucosa, replicating rapidly to establish dense biological niches along the respiratory tract (134) (**Figure 3**). These bacteria thrive in well-balanced ecosystems, reaching saturation above which competition for space and nutrients limits their growth (135–142). Under physiological conditions, an ecosystem characterized by high bacterial numbers and diversity promotes the utilization of a versatile pool of metabolites. It favors competition for nutrients (135–142), making it difficult for pathogenic bacteria to invade and colonize these niches. In diseased airways, deranged physiological conditions promote the overgrowth of specific bacterial species, decreasing community complexity and increasing preference for the consumption of particular metabolites (129). As a result, reduced competition for nutrients makes it easier for pathogenic bacteria to survive and colonize the respiratory mucosa (143, 144).

Similarly, the depletion of specific bacterial species, for instance, following prolonged antibiotic use, reduces pressure on the ecosystem, creating space and nutrients for other bacterial species to invade and colonize the mucosal field (145). Successful colonization and subsequent mucosal invasion by pathogenic micro-organisms trigger immune activation, inflammation, tissue damage, and further dysbiosis, creating a positive feedback loop that sustains airway inflammation. Evidence from several human airway microbiome studies summarized in a recently published multi-omics meta-analysis supports dysbiosis in inducing immune activation, inflammation, and tissue damage. Although the role of airway microbiome in COPD causation cannot be directly deduced, these findings show a difference in the bacterial communities in healthy and diseased states (24). During the early stages of COPD, airways are predominantly colonized with *H. influenzae, M. catarrhalis, and S. pneumoniae.* These bacterial species drive inflammation and tissue damage as previously described (111, 115–117, 119–121). Mechanisms of tissue damage induced by these bacteria are discussed further in the following sections. In advanced disease, impaired mucosal defense and reduced O_2_ tension in the airways favor the growth of *P.* aeruginosa, which promotes further inflammation and a vicious circle of tissue damage (146). Exposure and successful airway colonization by new strains of these bacteria trigger inflammation associated with exacerbation (108, 110, 146, 147) and impaired lung function (122).

Cell-to-cell communication between microbes also influences composition along the respiratory mucosa (134) (**Figure 3**). Interaction between different bacterial species along the airway mucosa determines microbial diversity with implications for COPD (108). Interspecies interactions have been observed among COPD patients colonized with bacterial species such as *H. influenzae, M. catarrhalis, S. pneumoniae, and P.* aeruginosa (110). Their composition significantly varies as individuals transition from stable disease to COPD exacerbation (147). In stable COPD, *S. pneumoniae* enrichment positively correlates with *H. influenzae* enrichment, while *P. aeruginosa* enrichment negatively correlates with *H. influenzae* and *M. catarrhalis* and positively correlates with *S. pneumoniae* enrichment (108). In the exacerbation state, the relationship between *S. pneumoniae* and *H. influenzae enrichment* disappears with the persistence of a correlation between *P. aeruginosa, H. influenzae, and M. catarrhalis* (108). These observations reveal extensive bacterial interactions among many unculturable bacterial species colonizing the airways. Most likely, this complex interaction is driven by underlying bacterial cell-to-cell communication as individual species establish their niche in the airways. Studies describing the molecular basis of this interaction in the airways are limited. A few illustrate bacterial interactions between selected species following nasopharyngeal colonization (110, 148–151). As described below, extensive studies performed on the gut microbiome illuminate these molecular mechanisms. We believe these pathways are shared. Gut microbiome studies have described bacterial species which directly inhibit other species within the same ecological niche through the secretion of antimicrobial peptides and lytic enzymes. Mechanistically, these factors impede the growth of adjacent bacterial cells *via* inhibition of peptidoglycan wall synthesis, pore formation, bacterial lysis, and nucleic acid degradation (152). A group of AMPs termed bacteriocins such as microcin, thuricin, and lantibiotics inhibit gram-negative bacteria such as *E.coli*, *S. enterica*, *C. difficile*, and *E. faecalis* (153–156). Other bacterial species deploy Type VI secretion system (T6SS) through quorum sensing to transport toxic cargo into their micro-environment or other bacterial cells (157–159). Consequently, this kills potentially pathogenic bacteria, maintaining a balanced ecosystem in the respiratory mucosa. For instance, the uptake of nucleic acids from commensal *Neisseria* species induces cell death of pathogenic *N. gonorrhea* (160). This is caused by the misrecognition of the DNA’s methylation pattern in *N. gonorrhea* (160). Similarly, the injection of a defective enzyme into a nearby bacterial cell by *P. aeruginosa* depletes the target cell’s ATP resulting in microbial cell death (161). *B. fragilis* secretes a ubiquitin-like protein *via* T6SS with potent inhibitory activity against coresident strains of *B. fragilis* (162). Besides secretion systems, bacteria utilize nanotubes to transport plasmids from bacterium to bacterium and extract nutrients such as iron from mammalian host cells (163, 164). As previously stated, a few airway microbiome studies have reported such complex bacterial ecosystem behavior. We hope that with the advent of enriched microbiology culture techniques such as mucin-enriched media, detailed investigations focusing on cell-to-cell interactions between airway bacterial species can be pursued. Such interactions occur in the respiratory mucosa shaping the airway microbiome. As elaborated previously, in diseased airways, the depletion of a single commensal species promotes the replication of another potentially pathogenic species. Disruption of such a balance in the ecosystem consequently triggers and sustains mucosal inflammation and bystander tissue damage (147) (**Figures 3–5**), as detailed in the following sections. This phenomenon has been described in COPD when the distal airway is colonized with bacterial species such as *P. aeruginosa*, depleting species such as *H. influenzae* and M. catarrhalis, and enriching other bacteria such as *S. pneumoniae*. We believe that the differential changes in the abundance of unculturable bacterial species inhabiting the airway following colonization with new strains or species occur, driving dysbiosis, which triggers acute inflammation associated with exacerbation (108, 110, 146, 147). Other possibly interacting bacteria that induce exacerbations, including *Chlamydia spp*, *L. pneumophilia*, *Mycoplasma spp*, and Non-typable *H. influenzae*, have been described elsewhere to drive immune inflammation (165)

## Microbiome-derived metabolites

A recent focus and comprehensive investigation of the microbiome metabolites, geared towards illuminating microbiome cell-to-cell communication and microbe-host cell immune crosstalk, has drastically improved our understanding of the mechanisms of microbiome mediated immune-modulation in health or dysregulation in disease. Microbial metabolites and their receptors have been described in detail elsewhere (80, 166, 167). Worth mentioning are metabolites of carbohydrates, amino acids, lipids, bile acids, and nucleic acids. In-depth characterization of microbial species based on their ability to produce specific metabolites shows that members of the *Bacteroidetes* phylum are good producers of acetate (C2) and propionate (C3). In contrast, Firmicutes are efficient producers of butyrate (C4). Other molecules, such as lactate and succinate, are converted to propionate (C3) by several species (168–173). C1-C4 molecules comprise the classical short-chain fatty acids (SCFAs). They can be transported systemically from their production site to other sites, for instance, from the gut to the liver, muscles, lungs, and brain, acting in an endocrine manner (80). These metabolites act on cognate G protein-coupled receptors (GPR43, GPR41, GPR109A, and Olfr78) (174, 175), widely distributed on several immune cells including but not limited to epithelial cells, neutrophils, macrophages, and dendritic cells to induce immune responses (176–180). Signaling *via* these GPRs generates pro-inflammatory responses *via* activation of MAP/PI3K kinases and mTOR (181). Anti-inflammatory responses are also generated when intracellular β-arrestins are engaged, inhibiting NF-kB (182–184). Both GPR41 and 43 promote proinflammatory responses, whereas only GPR43 can alternatively generate an anti-inflammatory signal (182–184). It is yet to become known which pathways are preferentially selected by SCFAs. To achieve an efficient immune modulatory program, phased engagement of both receptors would be expected to occur under physiological conditions. Failure of this to happen most likely, results in immune dysregulation, as could be the case for airway dysbiosis. As previously described, SCFAs induce epigenetic changes reprogramming mucosal immune cells to tolerate commensal bacteria.

In dysbiosis, SCFAs induce several defense mechanisms aimed at maintaining mucosal integrity, such as goblet cell activation, mucus hypersecretion (185, 186), replenishment of mucosal surface IgA levels (187–191), production of RegIIIγ (192) and NLRP3 inflammasome activation with the secretion of pro-inflammatory cytokines (193). Whereas these mechanisms have been demonstrated in the gut, parallel mechanisms could exist in the airways. Due to limited data supporting airway accumulation of SCFAs (194), the direct role of SCFAs on airway mucosal immune response cannot be deduced. Several investigators argued that peripheral immune cell activation and subsequent recruitment into the airways drove immune crosstalk (194). Indeed, several studies confirmed this hypothesis. Using animal models, authors demonstrated that the systemic effects of SCFAs were mediated *via* the bone marrow in a mechanism similar to trained immunity (195–199). In an allergic airway inflammation model, systemic acetate and propionate induced the production of macrophage and dendritic cell progenitor cells (MDPs) as well as common dendritic cell progenitor cells (CDPs) in the bone marrow, which subsequently migrated to the airways to form mature DCs (200–205). In another study, it was demonstrated that although the effects of SCFAs on bone marrow were context-dependent, they ultimately primed myeloid cell proliferation and subsequent migration into the airways, modulating immune responses in an airway influenza infection model (200, 206–208). The relevance of this crosstalk in the context of COPD pathogenesis is supported by evidence of the high prevalence of pulmonary impairment among individuals with chronic inflammatory bowel disease (50%) and individuals with irritable bowel syndrome (33%). Additionally, individuals with COPD are 2 to 3 times more likely to be diagnosed with irritable bowel disease and increased intestinal permeability (209–211). These observations warrant further investigation to underpin mechanisms of lung damage secondary to SCFAs.

Besides carbohydrate metabolism, other genera such as *Clostridium*, *Bacillus*, *Lactobacillus*, and *Streptococcus* metabolize amino acids including but not limited to glycine, lysine, arginine, leucine, isoleucine, and valine (212). Their biogenic derivatives, such as ammonia, monoamines, polyamines, histamines, agmatine, and cadaverine, induce immune responses *via* G protein-coupled receptors (213). Furthermore, the metabolism of branched-chain amino acids yields branched-short SCFAs such as isobutyrate, valerate, and isovalerate, which like C4 molecules, induce histone deacetylation, consequently regulating immune responses, as previously elaborated (214, 215). Of critical importance to immune activation and function is the molecule indole produced from tryptophan metabolism (216). Its derivatives, kynurenine, indole-3-acetic acid, and tryptamine, activate the aryl hydrocarbon receptor (AhR) (217), inducing ILC3 and Th17 immune responses (213, 218). A plethora of microbial species metabolizes bile acids as well. Bile acids induce immune cell responses *via* their receptors (FXR, VDR, PXR, and TGR5) (219, 220). Similarly, these metabolites induce several immune responses to preserve mucosal integrity (221–224) (73, 80, 200). In dysbiosis, metabolites are significantly altered, dysregulating immune activation networks locally and systemically with persistent inflammation. In COPD, this persistent inflammation drives tissue damage with reduced lung function and enhanced respiratory symptoms. Recently, several metabolites such as polyamines, xanthine, glycosphingolipids, and glycerophospholipids have been correlated with enhanced respiratory symptoms and reduced lung function (225). In another recent study, a microbiome-derived metabolic COPD signature comprising 46% lipid, 20% xenobiotic, and 20% amino acid-related metabolites has been reported (29). With recent advances in omics technology, there is a need to further characterize microbiome-derived metabolites as mediators of airway inflammation in COPD.

## Local versus systemic immune crosstalk: The role of the gut-lung axis

From the previous section, we can deduce that diverse metabolites’ activity drives the microbiome to impact immune responses locally and systemically. Several authors have referred to this crosstalk as the gut-lung axis to highlight the existence of communication between the gut microbiome and airway immune mucosal system as previously described (194, 226–230). Sufficient data indeed supports the role played by the gut microbiome in driving airway inflammation to cause COPD, as previously described (227, 231, 232). However, the mechanistic underpinnings of the gut-lung axis are yet to be fully deciphered in the context of COPD. Furthermore, whether a bidirectional lung-gut axis exists remains to be answered. Chronic airway diseases such as COPD, asthma, and cystic fibrosis show airway dysbiosis and induce gastrointestinal disturbances such as irritable bowel disease (227). However, this is most likely a result of oral intake of exogenous molecules such as antibiotics and other therapies for chronic respiratory diseases that ultimately cause gut dysbiosis and associated conditions. Mechanistic research utilizing parabiosis animal models is needed to decipher the gut-lung axis fully.

## Microbe-host immune interactions

Besides mucosal immune modulation *via* microbiome derived-metabolites, airway microbiome pathogen-associated molecular patterns (PAMPs) are sensed by the host pattern recognition receptors (PPRs) upon successful attachment to the mucosa (233) (**Figure 1**, **2**). Toll-like receptor TLR2, in conjunction with TLR1/6, recognizes bacterial lipoproteins, whereas TLR4 recognizes bacterial lipopolysaccharide (LPS) (234, 235). *Via* MyD88-dependent TLR signaling, mucosal epithelial cells secrete AMPs which inhibit bacteria by targeting cytoplasmic and cell-wall components (192, 236) (**Figures 1**, **2**). The significance of such a response has been demonstrated in mouse models where microbial depletion *via* antibiotic exposure significantly diminishes the mucosal secretion of AMPs (237), with a consequent reduction in the clearance of pathogenic bacteria (237). This is reversed upon mucosal replenishment with a bacterial cocktail, which activates epithelial cells to produce more AMPs (237). In addition to MyD88-dependent activation, sensing of microbial-derived products by nucleotide-binding oligomerization domain-containing receptors such as NOD1, NOD2, and NOD, LRR- and pyrin domain-containing 6 (NLRP6) inflammasome in the epithelial cells sets the pace for a pro-inflammatory signal along the mucosa (238–240). This response restricts mucosal colonization by pathogenic bacteria *via* secretion of IL-1β and IL-18 cytokines, which are processed from their precursors upon NLRP6 inflammasome activation (238) (**Figures 1**, **2**). In an animal model, authors showed by using mice deficient in IL-18 that activation of the NLRP6 inflammasome induced IL-18 secretion by epithelial cells and prevented gut colonization by pathobionts (238). A recent study has described a microbiome-mediated type I interferon immune response, dependent on cGAS-STING activation irrespective of MyD88-dependent signaling or direct host-bacteria interactions (241) (**Figures 1**, **2**). This study demonstrated that bacterial-derived outer membrane vesicles (OMVs) delivered into distal host cells activated the cGAS-STING-IFN-I axis, promoting clearance of both DNA and RNA viruses (241). Whether such findings can be applied to respiratory mucosa remains to be answered. Given similarities in embryonic origin, structure, and the immune responses mounted in the gut and airway mucosa (227, 242), we believe that a similar response is orchestrated along the airway to maintain mucosal integrity. Indeed, in one animal study, authors demonstrated the induction of a robust and broad innate immune protection of the airways following *S. pneumoniae* infection, effective against Gram-positive and Gram-negative bacteria and the fungus *A. fumigatus* (243). The response was characterized by activation of NF-κB, Type I/II IFNs, and Card9-Bcl-Malt pathways associated with upregulated expression of antimicrobial peptides (243). Amidst dysbiosis, sustained immune signaling results in aberrant immune response and inflammation along the mucosa, with bystander tissue damage. Earlier studies demonstrated the tissue-damaging effects of bacterial infection with non-typable *H. influenzae* using models of nasal turbinate epithelium (244). Similar observations have been recently reported for *N. subflava* (9). In dysbiosis, bacterial components such as lipoproteins, lipopolysaccharide, and peptidoglycans are released, inducing innate immune responses. These sustain inflammation and tissue damage associated with progressive obstruction observed in COPD.

## Mucins and the microbiome

The airway microbiome maintains the integrity of the mucus layer along the respiratory mucosa, preventing massive contact between microbes and the epithelial cells (134). Mucus along the respiratory airway is a major innate immune barrier that, to a significant extent, traps bacteria and clears the airway of these bacteria *via* mucociliary mechanisms (130). Goblet cells produce a variety of glycosylated mucin proteins along the mucosa (245). Mucins are secreted from the submucosal glandular and goblet cells lining the epithelium (246). Under homeostasis, the airway epithelial lining contains a few goblet cells and a moderate number of submucosal glands (247). Upon activation by bacterial products, environmental toxins, and proinflammatory cytokines, TNF-α, IL-1β, IFN-γ, IL-17A, IL-4, IL-9, and IL-13, goblet cells expand drastically, and submucosal glands increase in frequency, secreting several mucins (248). MUC5AC and 5B are the most predominant among mucin glycoproteins so far described, with MUC2 being the pioneer member characterized (248, 249). Further studies revealed that the gene encoding MUC2 is located next to the *MUC5A* on chromosome 11p15.5 (250), and its expression occurs early during goblet cell hyperplasia preceding that of the *MUC5AC* gene (248). Early studies demonstrated that bacterial-derived products such as LPS induced MUC2 expression *in vitro* using intestinal models *via* the Ras-MEK1/2-ERK1/2 signaling (251–253). Furthermore, the involvement of TLRs and NF-kB in mucin gene expression has been demonstrated (246). *H. influenzae* lysates upregulate MUC2 expression *via* the NF-kB activation (246). Other mechanisms reported to date include signaling *via* the TGF-β/Smad pathway (246).

Predominantly, mucin-2 is secreted in copious amounts on the mucosa following TLR2/4 and NOD1/2 activation (134). Together with other mucin proteins, mucin 2 forms an impervious layer that prevents massive contact between microbes and the epithelial cells. This barrier prevents potentially exaggerated immune responses. In the well-studied gut microbiome, bacterial species such as *B. thetaiotaomicron* and *F. prausnitzii* induce mucin gene expression, protein glycosylation, and mucus-secreting goblet cell differentiation (254). In addition to mucus production, *F. prausnitzii* maintains mucosal integrity by strengthening epithelial cell tight junctions in an IL-10-dependent manner (255, 256). Therefore, such bacterial species’ depletion reduces mucosal integrity and increases susceptibility to colonization by pathogenic bacteria (**Figure 5**). Once pathogenic bacteria successfully establish infection in the airways, mucus hypersecretion is induced as part of the acute inflammatory response (257–259). Early studies demonstrated some species, such as *H. influenzae*, *S. pneumoniae*, and *P. aeruginosa*, as potent mucin inducers (260). These studies further described *P. aeruginosa* as a proteolytic mucin-inducer in contrast to *H. influenzae* and *S. pneumoniae* as non-proteolytic mucin-inducers (260). Therefore, it is plausible that in the setting of dysbiosis predominated by known culprits (*H. influenzae, M. catarrhalis, S. pneumoniae, and P.* aeruginosa), sustained inflammation and mucin gene expression drives mucus production, compounded by impaired mucociliary clearance, distal airway occlusion, and reduced peak expiratory flow, typically observed in patients with chronic bronchitis (257–259). Mucin hypersecretion in COPD patients has been well-described in several studies (246, 261–270). Its accumulation in the airways of COPD patients has been reported as a predictor of mortality, especially among those with a lower baseline FEV_1_ (271). Mucus hypersecretion further reduces diffusion and exchange of gases across narrowed airways, worsening an already existing ventilation-perfusion mismatch. Furthermore, in dysbiosis, airway mucociliary activity is severely impaired, further complicating mucus clearance (111, 272–275). Mechanistically, bacterial pathogens induce direct epithelial cell injury, significantly disrupt mucociliary mechanics, and promote hypersecretion of mucus, which creates a conducive environment for pathogens to thrive (111, 272, 276–278).

## Antimicrobial peptides and lung damage in microbiome-immune crosstalk

As previously described, one of the outcomes of airway microbiome-immune crosstalk is the secretion of diverse cationic amphipathic peptides, known as antimicrobial peptides (279–281). These molecules kill invading bacterial pathogens in the mucosa, preserving microbial ecology (282, 283). In this review, we discuss two major classes of human-derived antimicrobial peptides: cathelicidins and beta-defensins (284, 285), and further highlight studies implicating such molecules in COPD pathogenesis (286, 287). We acknowledge the existence and role played by other lytic proteins, such as lysozymes (288–291), lactoferrin (292–294), secretory leucocyte protease inhibitor (295–298), cathepsins (299–303), granzymes (304–309), and S100 proteins (310–317). These molecules have been extensively discussed in the referenced articles.

The CAMP gene transcribes cathelicidin (LL-37) in mucosal epithelial cells and other cells of the immune system (318, 319). As elaborated elsewhere, LL-37 induces: (i) microbial killing *via* cell wall disruption (320–322), (ii) production of pro-inflammatory cytokines such as IL-1β, IL-6, and IL-17 (323–327), and anti-inflammatory cytokine IL-10, which suppresses IL-6, IL-12, TNF, HLA-DR, CD80, CD83, CD86, and CCR7 expression (326, 328, 329). Furthermore, LL-37 induces chemokines CCL2 and CCL7 in IFNγ-dependent manner (323) and polarizes macrophage differentiation towards classical (M1) phenotype and DCs towards cDC1s (330, 331). It also activates the inflammasome in macrophages and monocytes *via* the P2X7 receptor (332) and induces neutrophilic and eosinophilic migration (319). Other effects, such as angiogenesis, wound healing, and apoptosis, have also been reported (279). It is worth mentioning that, of all cells expressing LL-37, epithelial cells express the molecule only upon activation by inflammatory signals such as infection (318). Vitamin D3 has been demonstrated to regulate the expression of LL-37 *via* several vitamin D3 response elements located in the *CAMP* gene promoter region (333). Other than epithelial cells, Vitamin D3 regulates LL-37 expression in monocytes, keratinocytes, and neutrophils in an LPS-synergistic manner *via* TLR-1/2 in response to bacterial infection (319). Mechanistically, LL-37 binds and activates several extracellular and intracellular receptors (334), inducing pro-inflammatory genes *via* the activation of transcription factors, *NF-kB*, *CREB1*, *HIF1α*, *AP-1/2*, and *EGR-1* (326, 334, 335).

Besides the known protective effects of LL-37 against invading pathogens at the mucosa, several studies have implicated this peptide in COPD (289, 315, 336, 337, 337, 338, 338, 339, 339, 340, 340, 341, 341, 342, 342, 343, 343, 344, 344, 345, 345, 346, 346–351). Investigators have demonstrated its association with poor lung function (348, 349). In a setting of cigarette-smoke exposure, low levels of LL-37 have been independently associated with lower FEV_1._ This decrease in FEV_1_ is most significant among individuals with more deficient vitamin D3 (350) and is sustained at 6 and 18 months post-recruitment (351). Whereas low LL-37 was associated with a history of bacterial pneumonia, the inclusion of pneumonia in the adjusted model did not change the findings (350), implying a direct role of LL-37 in affecting lung function. It is still unclear whether increased susceptibility to bacterial infections sets the pace for inflammation and ensuing tissue damage in low LL-37 or whether the peptide directly impacts lung function. However, a recent study favoring the former demonstrates that inhibition of LL-37 promotes bacterial infection, dysbiosis, and inflammation (352). In contrast, earlier *in-vitro* studies tend to the latter, showing that endogenous cathelicidin (mCRAMP), a mouse ortholog, reduces emphysema severity, probably relating to its anti-inflammatory and healing features (353). Further investigation into the molecular mechanism of LL-37-mediated-COPD pathogenesis is urgently needed. In the setting of well-established COPD, the profile of LL-37 completely reverses, with several studies reporting elevated levels in COPD compared to healthy controls (349, 354–360). This peptide is further elevated during acute COPD exacerbations compared to stable disease due to inflammation secondary to bacterial infection (296), supported by elevated pro-inflammatory cytokines, IL-6, and IL-8 (296). Moreover, in stable COPD, high levels of LL-37 predict acute exacerbation (296, 361). As expected, COPD patients with vitamin D deficiency have significantly lower LL-37 (362–365). This association is even more evident among individuals with acute exacerbation (296, 361). Complex changes in antimicrobial peptides have been reported in dysbiosis among individuals with. For instance, following infection of the airways with a new bacterial strain, LL-37 increases, while lysozyme and SLPI decrease (289). These changes in LL-37 and SLPI are more significant in exacerbation compared to the stable disease (289). Within COPD stages, LL-37 significantly varies from one study to another. Several investigators have reported conflicting results of LL-37 levels based on COPD severity stages (366). Most studies to date have been limited by small sample sizes, heterogeneous sampling (induced versus expectorated sputum versus BAL fluid), and COPD heterogeneity (smoking versus non-smoke-related COPD). This warrants the careful design of adequately powered studies investigating LL-37 as a COPD severity biomarker. Nevertheless, a few studies on COPD stratification based on exacerbation risk shows that the LL-37 is lower in high-risk exacerbators compared to low-risk counterparts (361, 366, 367). Similarly, COPD stratification based on severity shows that LL-37 is significantly reduced in severe disease compared to mild/moderate disease (296, 348). Several authors argue that this observation is most likely driven by a high background of NF-kB activation in severe disease, which has been reported to inhibit LL-37 (366).

A second family of antimicrobial peptides, called defensins, exists and contributes to immune protection against pathogenic bacteria, as described elsewhere (283). Two classes, α- and β-defensins, exist based on the length of their peptides (368, 369). These molecules contribute to granulocyte antimicrobial activity, intestinal mucosal cell defense, and cutaneous host defenses. Six members of the α-defensin include neutrophil peptides (type1-4 α-defensins) secreted by neutrophils, monocytes, lymphocytes, and NK cells. At the same time, types 5 and 6 are expressed and secreted by Paneth cells of the small intestines, epithelial cells of the airways, and gastrointestinal and female reproductive tracts (370). In contrast, β-defensins are widely distributed and expressed in epithelial cells, monocytes, macrophages, and DCs (370). Whereas type 1 β-defensin is constitutively expressed in epithelial cells, type 2 β-defensin is inducible by NF-kB activation (370). Bacteria in the mucosa stimulate the production of both type 3 and 4 β-defensins (371, 372). TNF has also been reported to stimulate the production of type 3 β-defensins. Like LL-37, the secretion of β-defensins produces both pro- and anti-inflammatory signals. For instance, upon activation, neutrophils secrete α-defensins which increase the expression of TNF and IFNγ, with a consequent increase in the expression of FcγRIIB and FcγRI (373). These changes ultimately enhance macrophage phagocytosis. On the contrary, activation of necroptotic neutrophils induces high levels of α-defensins which antagonize the release of nitric oxide and pro-inflammatory cytokines from macrophages, toning down the pro-inflammatory signals (374). Other effects described elsewhere include chemotaxis, angiogenesis, wound healing, antitumor, antifungal, antiviral, and less potent antimicrobial activity (279). β-defensins have been implicated in COPD pathogenesis and disease progression (297, 315, 375–381). Reduced lung function has been associated with increased levels of defensins (380, 382–384). Earlier studies investigating mechanisms of tissue damage in COPD by neutrophils demonstrated that upon neutrophilic infiltration of the airways and activation, neutrophils release defensins which not only play a role in antimicrobial defense but also induce lung tissue injury in addition to other molecules such as serine elastases, cathepsin G and proteinase 3 (375). In *in vitro* models, these molecules decreased the integrity of the epithelial cell layer and frequency of ciliary beat, as well as inducing mucus hypersecretion and mediating immune modulation (375, 385). The activity of defensins is known to be regulated by the α1-proteinase inhibitor. This molecule competitively blocks defensin’s cytotoxic and stimulatory activity toward epithelial cells (375).

## Macrophages and the microbiome

Airway microbiota has been demonstrated to mobilize and reprogram alveolar macrophages, as illustrated in **Figures 1**, **2**. In antibiotic-treated mice, depletion of commensal bacteria induced lower frequencies and numbers of alveolar macrophages. Specifically, these macrophages were reprogrammed to express higher levels of Arg1, CCL24, IL-13, IL-10, IL-6, and IL-1β, consistent with the M2 phenotype (386). These macrophages returned to normal levels following the administration of a consortium of commensal bacteria in the respiratory mucosa of animals previously treated with antibiotics (386). In addition, the microbiome has been shown to potentiate macrophage-bacterial killing and clearance *via* granulocyte-macrophage colony-stimulating factor (GM-CSF) signaling, reported to be driven by the IL-17/22 axis (387). Models of commensal colonization in antibiotic-treated and germ-free mice have shown that potent NLR-stimulating bacteria in the upper airways promote resistance to airway mucosal colonization by pathogenic bacteria through NOD2 and GM-CSF signaling (387). Antibiotic treatment has also been associated with impaired alveolar macrophage metabolism and defective bactericidal activity (388). Whole-genome mapping of alveolar macrophages revealed upregulation of metabolic pathways in dysbiosis (389), correlating with reduced cellular responsiveness (389). Compared with controls, alveolar macrophages obtained from microbiome-depleted mice showed a diminished capacity to phagocytose bacteria (389). In dysbiosis, pathobionts directly infect alveolar macrophages and use them as vehicles to access the lung interstitium (**Figures 3**, **4**), attracting neutrophils, monocytes, and other inflammatory cells to orchestrate inflammation and lung damage (9, 390). In dysbiosis, the airway microenvironment drives the activation of alveolar macrophages, resulting in mixed phenotypes observed in COPD (391). Colonizing the distal airways with pathogenic bacteria sets an inflammatory tone characterized by increased levels of pro-inflammatory cytokines such as TNF-α and IL-6 (391). The induction of M1/M2 macrophage phenotypes results in a mixed phenotype of airway inflammation and tissue damage driven by classical (i.e., M1) phenotype and airway remodeling and fibrosis driven by alternative (i.e., M2) phenotype. Similar to smoke-induced COPD, other studies report a gradual transition from upregulation of M1 genes to downregulation of the same genes followed by upregulation of M2-related genes (9, 392), which is usually a typical pattern followed from acute inflammation to resolution. Chronic inflammation driven by dysbiosis with a resultant mixed M1/M2 phenotype is the most likely occurrence. M1-driven lung damage is mediated *via* matrix metalloproteinase (MMP) activity, oxidative stress caused by reactive oxygen and nitrogen species, and many other lytic proteins on parenchymal tissue as elaborated elsewhere (2, 4, 393–396).

## Neutrophils and the microbiome

Whereas airway-based human studies demonstrating the direct impact of the microbiome on airway neutrophilic responses are currently scarce, much evidence is inferred from data obtained from intestinal mouse models, which have been extensively reviewed and well-elaborated elsewhere (397). Among highlighted studies, the microbiome has been demonstrated to modulate the neutrophil function and aging *via* TLR- and MyD88-mediated signaling pathways (398). In a germ-free mouse model, the inflammatory challenge was associated with a reduction in neutrophil recruitment and cytokine production (399), which reversed in the setting of lipopolysaccharide pre-treatment. Notably, this arrest in neutrophilic migration and function was IL-10 dependent (399). In another study, depletion of the microbiome significantly reduced the quantity of circulating aged neutrophils, thus considerably reducing neutrophil-mediated tissue damage (398).

Furthermore, the microbiome has been reported to influence the development and function of neutrophils in several micro-environments (75). Following the development, immature neutrophils demonstrate limited functional quality characterized by reduced proinflammatory activity. However, after microbial sensing *via* MyD88-dependent pathways, neutrophils become better at phagocytosis and execution (**Figure 4**) (400, 401). In germ-free and antibiotic-treated mouse models, neutropoiesis in bone marrow is severely impaired, consequently delaying systemic bacterial clearance (401). Similarly, microbiome-derived metabolites such as short-chain fatty acids (SCFAs) drive neutropoiesis *via* similar mechanisms as previously described (200). In another study, the same metabolites and cell-wall components were demonstrated to induce IL-17 cytokine production from type 3 innate lymphoid cells (ILC3s) and IL-17-producing memory mucosal CD4+T cells, consequently ramping up neutropoiesis *via* G-CSF production (402). A neutrophilic response at the airway mucosa is critical to eliminate pathogenic micro-organisms, which overcome colonization resistance and successfully invade the mucosal barrier. Airway dysbiosis activates neutrophils *via* the MyD88-dependent pathway, resulting in NF-***K*** B and MAPK signaling in airway epithelial cells (403–406). Through NETosis (407–409), activated neutrophils release serine-proteinases such as elastases, cathepsin G, and proteinase-3 from primary azurophilic granules. These proteinases cleave elastin, causing lung tissue damage observed in COPD (395, 410–412). Furthermore, secondary and tertiary granules release two metalloproteinases, i.e., collagenases (MMP8) and gelatinases (MMP9), which degrade lung extracellular matrix (395). Persistent neutrophil activation and NETosis orchestrate bystander tissue damage (407–409). (**Figure 4**, **5**). In dysbiosis, LPS and TNF-α induce the production of neutrophilic chemoattractants by activated epithelial cells such as CXCL1 and CXCL8 (413), which promote neutrophilic infiltration into the airways, further driving neutrophilic inflammation and tissue damage (413). As discussed in the proceeding sections, Th17 activation further augments neutrophilic inflammation and tissue damage (414, 415).

## Innate lymphoid cells and the microbiome

The innate lymphoid cell family is comprised of cytotoxic cells (the natural killer cells) and non-cytotoxic subsets (referred to as ILC1, ILC2, and ILC3, based on similar nomenclature as T helper cellular sub-phenotypes) (75). Whereas studies investigating the microbiome’s influence on ILCs within the airway mucosa are few, the effect of the microbiome on ILC2 and ILC3 cells has been highlighted (416). In one study, intranasal administration of OM-85 bacterial lysate abrogated experimental allergic asthma by targeting IL-33/ILC2 axis (97). In the next section, we focus on findings from the mouse gut to infer mechanisms influencing ILC-specific immune responses along the airway mucosa. Studies examining the microbiome’s influence on ILCs have concentrated mainly on ILC3s. Recently published reports show that the depletion of ILC3s in mouse models significantly abrogates IL-22 production, with consequent loss of bacterial control within the mucosa, resulting in disease (417). In another study, authors characterized the effect of the gut microbiome on ILC regulatory landscape using antibiotic intervention and germ-free mouse models at the single-cell level. ILCs differentially integrate signals from the microbial microenvironment and generate phenotypic and functional plasticity (418). Furthermore, *via* ILC3 activation, the microbiome modulates the activity of other immune cells within the mucosa. For instance, (i) microbiome induces commensal-specific CD4+T cells to maintain tolerance at the mucosa (419, 420); (ii) Microbial sensing, inflammasome activation, and the production of IL-1β by macrophages drives GM-CSF secretion by ILC3s, required for macrophage function and induction of tolerance (421); (iii) the production of TNF-β by activated ILC3s drives IgA production at the mucosa (422). (iv) Finally, microbial induction of IL-22 production by ILC3s induces the expression of antimicrobial peptides from epithelial cells required for mucosal host defense (423). It is, therefore, plausible that dysbiosis at the airway mucosa disrupts ILC3-mediated immune regulation, consequently driving aberrant Th17-mediated damage (424) (**Figure 5**). Although the literature on ILCs in COPD is still minimal (425, 426), the role of ILC3s in COPD warrants investigation since IL-17, its principal cytokine, has already been described as a known driver of neutrophilic inflammation, and individuals with COPD have increased levels of IL-17 (426, 427). Furthermore, IL-17, IL-22, and IL-23-expressing immune cells have been reported in bronchial biopsies from COPD patients (428). Gene expression analysis in lung tissue from COPD patients further provides evidence for the role of ILC3 in COPD (429).

## Dendritic cells and the microbiome

Dendritic cells (DCs) are strategically positioned within the airway mucosa, residing in mucosa-associated lymphoid tissue (MALT), such as the Welder’s ring in the upper airways, bronchial lymph nodes in the distal airways, as well as multiple satellites within the lamina propria along the airway mucosal tract (430, 431). Using their dendrites, DCs continuously sample airway bacteria that attach and colonize the mucosa (432–435) or gain access to the MALT *via* the epithelial tight junction barrier (431, 436–438). Extensive work reviewed elsewhere (431, 438, 439) suggests that depending on the type and degree of microbial exposure, airway DCs induce a wide range of immune responses from immune tolerance induced by plasmacytoid DCs to inflammation induced by conventional DCs (cDCs) (439). Phenotypic characterization of the microbiome based on DC immune responses is still a work in progress. In a recent study investigating the effect of airway microbiome-derived bacterial strains on DC activation, CD4+T cell priming, and cytokine response (440), *P. aeruginosa* induced high levels of TNF-α, IL−12, and IL-6 from DCs and primed CD4+T cells to secrete IFN-γ and IL-22 compared to *S. pneumoniae* and *R. mucilaginosa* (440). In another study, it has been confirmed that *R. mucilaginosa* inhibits airway immune pro-inflammatory responses *via* NF-κB-dependent mechanisms (38). This evidence illustrates the impact of dysbiosis on mucosal DC activation and eventual T-cell licensing required to orchestrate inflammation and tissue damage. Studies describing how activated DCs induced by dysbiosis drive COPD pathogenesis are minimal or still a work in progress. The role of activated DCs in driving COPD pathology has been described in the context of cigarette smoke-induced COPD (441–445), which is outside the scope of this review. As described above, bacterial species such as *P. aeruginosa*, known to predominate in advanced COPD, activate DCs, inducing high levels of TNF-α, IL−12, and IL-6, priming CD4+T cells to secrete IFN-γ and IL-22 (440). Whereas activated DCs secondary to cigarette smoke exposure have been reported to skew adaptive immune response towards Th1 and cytotoxic T cell lymphocyte (CTL) responses (known hallmarks of chronic inflammation in COPD) (442), currently, it’s not known how dysbiosis induces DCs into either type 1 or 2 DCs, which ultimately dictates CD4+T cell phenotypes.

## T cells and the microbiome

As previously described, continuous microbial sampling and trafficking by activated DCs, and alveolar macrophages deliver processed microbial antigens to naïve CD4+T and CD8+T cells within MALT and draining lymph nodes (439) (**Figures 4**, **5**). Following microbial-driven immune sensing and activation, the production of IL-12, IL-23, IL-27, and notch ligand by airway DCs, alveolar macrophages, and epithelial cells induces a Th1 response that regulates mucosal colonization by potentially pathogenic bacteria (439). The microbiome is required for optimal mucosal T-cell development, function, and memory. This role has been demonstrated in intestinal mouse models where germ-free mice have been observed to have developmental defects in lymphoid tissues (446, 447). Specifically, these animals display reduced frequencies of mucosal CD4+ and CD8+T cells and decreased numbers of TCR-expressing intraepithelial lymphocytes. Among CD4+T cell phenotypes, microbiome-mediated mucosal inflammation has been strongly linked to aberrant Th17 and suppressed Treg responses in COPD. For instance, enrichment of the lung microbiome with oral taxa is associated with Th17 lung inflammation in COPD (39). Similarly, in bleomycin-induced mouse interstitial pulmonary fibrosis (IPF), airway dysbiosis induces IL-17 cytokine, which ameliorates following either specific airway bacterial depletion or IL-17 blockade. Three commensal bacteria belonging to the genera Bacteroides and Prevotella promote fibrotic pathogenesis through MyD88-dependent IL-17R signaling *via* bacterial exosomes (448). These findings have been replicated in human studies where *Prevotella* and *Veillonella* spp have been associated with enhanced Th17 inflammatory response in COPD (449). In another animal-based study, *S. mitis*, *V. parvula*, and *P. melaninogenica* induced dysbiosis-mediated inflammasome and Th1/Th17 activation with reduced susceptibility to *S. pneumoniae.* These data imply an immunoprotective role of specific bacterial species in the airways (40). However, chronic Th17 inflammation in persistent dysbiosis promotes lung tissue damage (448). Besides Th17, microbial interaction with mucosal CD4+T cells induces immune tolerance. This process involves several mechanisms extensively described elsewhere (450). We highlight some of these mechanisms here. MyD88-dependent TLR2 activation by microbial-derived PAMPs, such as capsular polysaccharide A from *B. fragilis*, has been shown to induce the expansion of Foxp3+Tcells within the mucosa (451). Foxp3+T cells drive IL-10 production, facilitating mucosal immune tolerance. *B. fragilis* have also been shown to deliver antigenic products through bacterial exosomes, which upon phagocytosis by host immune cells *via* a non-canonical autophagy pathway, induce IL-10 expressing Foxp3+T cells (450, 452). In another mechanism, microbial-induced Tregs promote mucosal memory B or plasma cells’ IgA secretion, epithelial cells’ tight-junction protein expression, and goblet cells’ mucus production in an IL-10-dependent manner (453, 454). This maintains microbial biomass and diversity and facilitates Treg expansion through a symbiotic regulatory loop, preventing overt inflammation (450). Short-chain fatty acids potentiate Foxp3+ cell differentiation and immunomodulatory activity in the gut mucosa as previously discussed (87–89, 455). This evidence implies a tight regulatory role played by mucosal commensal bacteria in maintaining a robust mucosal Treg response. In dysbiosis, however, a heightened Th17 immune response increases TGF-β production with consequent Treg downregulation (456, 457).

## B cells, mucosal surface IgA, and COPD

A recently published article describes the role of the microbiome in shaping B-cell immune responses (63). The authors discuss microbiome-driven B cell immune activation, antibody class switching from IgM to IgA, and maintenance of memory B or plasma cells at the mucosal surface (63, 458–460). Although studies referenced in this article are biased toward the gut mucosa, emerging evidence from airway studies confirms the engagement of similar immune responses (461). Upon antigenic encounter at the mucosa, naïve B cells, *via* antigen-specific B cell receptors, engage the antigen, inducing an activating signal. B cells proliferate and undergo clonal selection, affinity maturation, and class switching within mucosal lymphoid tissue and some in lymph nodes, producing high-quality IgA antibodies (462). This process largely depends on T follicular helper cells (461). A recent study revealed increased expression of the cytokine IL-21 among patients with COPD, primarily in CD4+T cells. IL-21 promotes B cell maturation, antibody affinity maturation, and differentiation into plasma cells. Without co-stimulation, B cells exposed to IL-21 undergo apoptosis which controls bystander B cell immune activation (463). In addition to T cell-dependent help, evidence shows that bacterial products such as LPS can induce human IgM+ B cells to directly class switch to IgA-secreting plasma cells (461). T-independent induction of IgA has been shown to occur in isolated lymphoid follicles and the mucosal lamina propria, generating polyreactive IgA with low affinity for commensal bacteria (462). Upon production by plasma cells, IgA is transported across the mucosal epithelial cells *via* transcytosis and secreted onto the mucosal surface in a process that requires the binding of dimeric IgA to the polymeric IgA receptor, at least in intestinal models (464). Mucosal surface IgA preserves microbial ecology at the mucosa in several ways. It binds and enchains dividing bacteria, limiting their association with the epithelial cells (465). Selective binding of IgA to some bacterial species also inhibits bacterial cell growth. Studies have demonstrated the induction of sustained commensal-specific IgA memory responses, which become attenuated when a new bacterial strain or species invades the mucosa (458). This evidence implies a continuous IgA repertoire modification to match the dynamic changes in the mucosal microbial communities. This is possible *via* re-entry and further affinity maturation by somatic hypermutation of existing B cell memory clones in the mucosal lymphoid tissue (466). Bacterial coating with IgM and IgG has also been demonstrated in human intestinal models. Specifically, IgM-secreting plasma cells recognize mucus-dwelling commensals (467). Without IgA, a compensatory IgM response ensues to contain the bacteria (467). It is worth mentioning that microbiome-specific IgA, IgM, and IgG antibodies produced locally can act systemically in similar mechanisms as already described in the gut-lung axis (468). Microbiome-derived metabolites also modulate B cell immune function *via* induction of epigenetic changes *via* HDAC inhibitory activity, which induces histone acetylation, enhancing gene expression necessary for B cell differentiation into IgA/G secreting plasma cells (189, 469).

Following the proposition that autoimmunity significantly contributes to COPD pathogenesis, investigators have concentrated their efforts on underpinning the role of B cell immune responses in COPD (470–475). Earlier studies demonstrated the presence of lung lymphoid follicles and elastin-specific antibodies among patients with advanced emphysema (476, 477). Furthermore, unbiased gene expression analysis among emphysema patients revealed a strong link between airway B cells and emphysema (478). This preliminary data provided evidence to investigate further the mechanistic role of B cells in COPD pathogenesis. Studies have additionally demonstrated B cells’ critical role in promoting COPD immunopathology (479–491). For instance, enhanced B cell differentiation into IgA-producing plasma cells has been demonstrated in the airways of COPD patients, where the bronchial epithelial cells primarily provide B cell differentiating signals *via* the IL-6/IL-6 receptor and BAFF-APRIL/TACI pathways (492). IgA-producing B cells are increased in the distal airways of COPD patients compared to healthy controls and positively correlate with COPD severity scores (461). Results are similar in animal models following infection with *P. aeruginosa*, a known pathogen in COPD (461). Mechanistically, the direct role of mucosal IgA in inducing lung damage has not yet been fully elucidated (493). The non-inflammatory nature of IgA highly suggests that this molecule is less likely to contribute to COPD pathogenesis directly. However, evidence from known pathological consequences of IgA, such as IgA nephropathy (494), warrants further investigation into the direct role of IgA in COPD pathology. In airway dysbiosis, increased mucosal surface IgA is associated with airway inflammation in COPD (495–499).

## Conclusion

This review highlights numerous airway microbiome-mediated immune pathways, mostly in animal models, that drive COPD pathogenesis. Such responses are characterized by alveolar macrophage, dendritic cell, neutrophil, monocyte, innate lymphoid cell, and Th1/Th17 cell activation phenotypes whose interaction with airway epithelial cells culminates into sustained lung inflammation and tissue damage. Although a few human-based COPD studies have also been highlighted, more research is needed to test and validate these findings in human COPD cohorts. Immunophenotyping microbiome habitats has the potential to advance microbiome-based therapeutics. Microbiome-resulting local immunophenotypes are, however, thus far poorly characterized. Accordingly, deep immune phenotyping of the airway host-microbiome interface, possible through multi-omics approaches, may meaningfully inform far more precise interventions in COPD (500).

## Author contributions

AK: Conceptualization, methodology, original draft writing, editing, and funding acquisition. NMR, MN, JN: Draft review and editing. TS, MJ, HM-K, BB, OJS, BK, and SF: Mentorship, funding acquisition and drafting the manuscript for important intellectual content. All authors contributed to the article and approved the submitted version.
